# Clinical outcomes at 1 year in early Psoriasis Area and Severity Index responders compared with non‐responders: Subgroup analysis of UNCOVER‐3 trial

**DOI:** 10.1002/ski2.43

**Published:** 2021-05-11

**Authors:** D. Rosmarin, S. Smith, D. Shrom, R. Burge, K. See, M. McKean‐Matthews, T. Ridenour, C.‐Y. Lin, J. Gorelick

**Affiliations:** ^1^ Tufts Medical Center Boston Massachusetts USA; ^2^ California Dermatology and Clinical Research Institute Encinitas California USA; ^3^ Eli Lilly and Company Indianapolis Indiana USA; ^4^ Division of Pharmaceutical Sciences University of Cincinnati Cincinnati Ohio USA; ^5^ Syneos Health Morrisville North Carolina USA; ^6^ California Skin Institute San Jose California USA

## Abstract

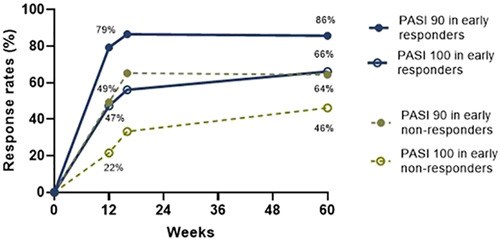

## Dear Editor,

Alignment of patient needs and treatment goals with treatment choices is imperative for patient‐oriented psoriasis care. Rapid response is a major treatment goal from a patient's perspective.^1^ Patients desire substantial improvement as early as 2 weeks and, subsequently, complete resolution.^2^ With the advent of newer biologics, such as interleukin‐17 inhibitors, rapid efficacy (Psoriasis Area and Severity Index [PASI]‐75/90 responses) can be achieved within 2 weeks of starting treatment^3^; however, the impact of rapid responses on long‐term outcomes is unclear. We investigated the impact of 50% improvement (PASI‐50) as early as Week 2 on PASI‐90 and PASI‐100 response rates at Weeks 12 and 60, using data from the UNCOVER‐3 (NCT01646177) trial of ixekizumab (IXE), a high‐affinity monoclonal antibody that targets interleukin‐17A, in patients with moderate‐to‐severe psoriasis.^3^

In UNCOVER‐3, patients received IXE 80 mg once every 2 weeks (IXEQ2W) or every 4 weeks (IXEQ4W) after a 160 mg starting dose, etanercept 50 mg twice weekly or placebo for 12 weeks.^3^ During the long‐term extension, all patients received IXEQ4W until Week 60. Patients initially assigned to etanercept had a 4‐week placebo washout before switching treatment.^3^ This analysis includes patients initially randomized to IXEQ2W (*N* = 385), now the commercially approved dose, with the Week 60 analysis focusing on patients who entered the long‐term extension.

Within treatment groups, patients were classified as early responders (Week 2 PASI‐50) or early non‐responders (not achieving PASI‐50 by Week 2). Subgroups were examined for PASI‐90 and PASI‐100 responses at Weeks 12 and 60; response rates were compared between early responders and early non‐responders using within‐treatment Chi‐square tests. Missing percentages were 5.2% (Week 12) and 7.5% (Week 60). Non‐responder imputation was used. One‐year discontinuation rates were examined.

Baseline characteristics, including age, psoriasis duration, static Physician's Global Assessment and PASI scores, were similar between subgroups (Table S1). The 6.5 kg difference in mean body weight between groups was not powered for significance. Among Week 2 responders (241; 62.6%) and non‐responders (144; 37.4%), baseline mean (standard deviation [SD]) PASI scores were 20.7 (8.0) and 20.8 (8.5), respectively. Week 12 mean (SD) PASI values were 1.04 (1.98) (early responders) and 3.50 (5.71) (non‐responders); *p* < 0.001. Week 60 mean (SD) PASI values were 0.75 (1.81) (early responders) and 2.17 (4.43) (non‐responders); *p* = 0.076.

**FIGURE 1:**
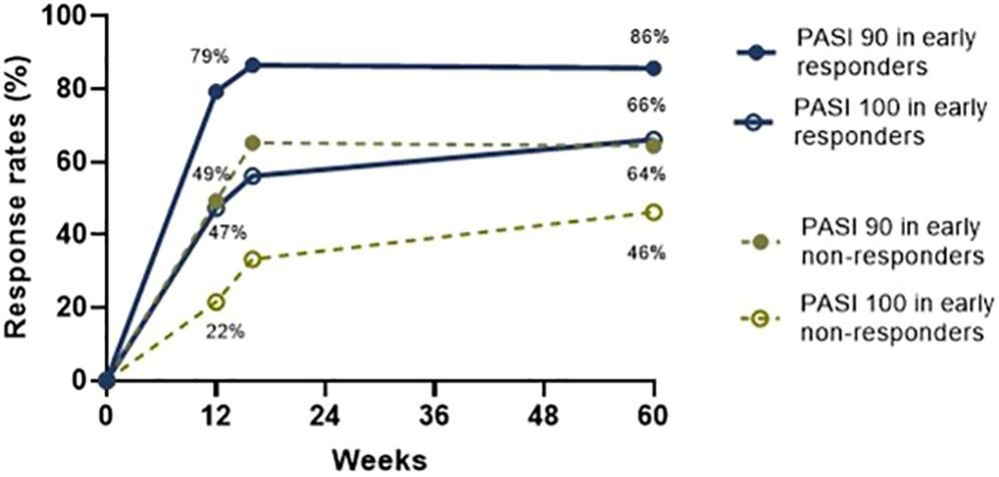
Response rates at 1 year in ixekizumab (once every 2 weeks) early responder and non‐responder subgroups. Early responder refers to patients who achieved PASI‐50 response at Week 2 and early non‐responders refers to those who did not achieve PASI‐50 response by Week 2. PASI, Psoriasis Area and Severity Index

Week 12 PASI‐90 rates were significantly higher in early IXE responders (79.3% [95% CI 74.1%, 84.4%]) versus non‐responders (49.3% [95% CI 41.1%, 57.5%; *p* < 0.001]); PASI‐100 rates were also significantly higher in early IXE responders (47.3% [95% CI 41.0%, 53.6%; ]) compared with non‐responders (21.5% [95% CI 14.8%, 28.2%; *p* < 0.001]; Figure 1). Week 60 response rates were significantly higher among early responders (PASI‐90: 85.7% [95% CI 81.1%, 90.2%]; PASI‐100: 66.1% [95% CI 60.0%, 72.2%]) compared with early non‐responders (PASI‐90: 64.4% [95% CI 56.2%, 72.6%]; PASI‐100: 46.2% [95% CI 37.7%, 54.7%]; *p* < 0.001; Figure 1). One‐year discontinuation rates were lower among early responders (8%) versus early non‐responders (18%; *p* = 0.005).

PASI response rates were greater at Weeks 12 and 60 in patients with PASI‐50 at Week 2 compared with non‐responders. A head‐to‐head study comparing IXE and ustekinumab reported rapid skin improvement with IXE as early as Week 2 as an important predictor for maintaining PASI‐90 and PASI‐100 responses over 52 weeks.^4^ In IXORA‐S, PASI‐50 at Week 2 was a significant predictor for PASI‐100 responses (*p* = 0.04) over 52 weeks. More Week 2 responders maintained long‐term PASI‐90 and PASI‐100 response rates and more Week 2 responders maintained long‐term PASI‐90 and PASI‐100 response rates with IXE versus ustekinumab.^4^ Additionally, previous research demonstrated rapid reduction of itch following IXE treatment which preceded clinically meaningful PASI improvements in most patients.^5^ IXE was associated with faster itch reduction within a week (vs. etanercept), with subsequent PASI improvements at Week 12, indicating greater skin improvement than etanercept.^5^ Prior reports also suggest that patients treated with IXE achieved faster, more pronounced PASI response versus ustekinumab, and higher levels of skin clearance were associated with improved long‐term outcomes.^6^

Our results were similar. Patients with early Week 2 PASI response had higher and sustained PASI response rates at 1 year. At Week 2, 62.6% of IXE‐treated patients were PASI‐50 responders, with 68.5% of early responders achieving Dermatology Life Quality Index (0,1) at Week 12. Lower discontinuation rates noted among responders compared with non‐responders further highlight the importance of rapid treatment responses. Subgroup analyses support superior drug survival with IXE versus adalimumab and secukinumab.^7^, ^8^

In conclusion, early treatment responses were associated with greater long‐term response rates and could be an important predictor of long‐term psoriasis outcomes. These findings are meaningful as they assess the impact of an important treatment goal from the patients' perspective on long‐term outcomes.

## CONFLICTS OF INTEREST

D. Rosmarin has received honoraria as a consultant for AbbVie, Celgene, Dermavant, Dermira, Eli Lilly and Company, Incyte, Janssen, Novartis, Pfizer, Regeneron, Sanofi, Sun Pharmaceuticals and Viela Bio; research support from AbbVie, Amgen, Bristol Myers Squibb, Celgene, Dermira, Eli Lilly and Company, Galderma, Incyte, Janssen, Merck, Novartis, Pfizer and Regeneron Pharmaceuticals Inc; and has served as a paid speaker for AbbVie, Amgen, Celgene, Eli Lilly and Company, Janssen, Novartis, Pfizer, Regeneron Pharmaceuticals Inc. and Sanofi.

## Supporting information

TABLE S1
