# Supplementary material for: Clinical outcomes at 1 year in early Psoriasis Area and Severity Index responders compared with non‐responders: Subgroup analysis of UNCOVER‐3 trial
Source: Skin Health Dis. 2021 May 11;1(3):e43. doi: 10.1002/ski2.43 (PMC9060090; doi:10.1002/ski2.43)
Supplement: Supplementary file 1 — TABLE S1 [file SKI2-1-e43-s001.docx]

**Table S1. Selected baseline demographics and clinical characteristics for Week 2 PASI-50 responders and non-responders**

| **Parameter** | **IXE80Q2W**  **Week 2 Responders**  **(n=241)** | **IXE80Q2W**  **Week 2 Non-responders**  **(n=144)** |
| --- | --- | --- |
| Age, years | 44.8 (13.7) | 46.9 (12.0) |
| Weight, kg | 87.9 (22.9) | 94.4 (23.8) |
| Duration of psoriasis symptoms, years | 18.4 (11.8) | 16.8 (12.8) |
| sPGA | 3.5 (0.6) | 3.6 (0.6) |
| PASI score | 20.7 (8.0) | 20.8 (8.5) |
| Prior biologic thereapy, n (%) |  |  |
| Never used | 198 (82.2) | 129 (89.6) |
| Ever used | 43 (17.8) | 15 (10.4) |

Data are presented as mean (SD) per responder and non-responder groups, unless specified otherwise.

IXE80Q2W, ixekizumab 80 mg once every 2 weeks; PASI, Psoriasis Area Severity Index; sPGA, static Physician’s Global Assessment.
